# Mutual exclusion of *Asaia* and *Wolbachia* in the reproductive organs of mosquito vectors

**DOI:** 10.1186/s13071-015-0888-0

**Published:** 2015-05-17

**Authors:** Paolo Rossi, Irene Ricci, Alessia Cappelli, Claudia Damiani, Ulisse Ulissi, Maria Vittoria Mancini, Matteo Valzano, Aida Capone, Sara Epis, Elena Crotti, Bessem Chouaia, Patrizia Scuppa, Deepak Joshi, Zhiyong Xi, Mauro Mandrioli, Luciano Sacchi, Scott L. O’Neill, Guido Favia

**Affiliations:** Scuola di Bioscienze e Medicina Veterinaria, Università di Camerino, 62032 Camerino, MC Italy; Dipartimento di Scienze Veterinarie e Sanità Pubblica, DIVET, Università degli Studi di Milano, Milan, Italy; Dipartimento di Scienze per gli Alimenti la Nutrizione e l’Ambiente, DeFENS, Università degli Studi di Milano, Milan, Italy; Department of Microbiology and Molecular Genetic, Michigan State University, Michigan, USA; Dipartimento di Scienze della Vita, Università di Modena e Reggio Emilia, Modena, Italy; Dipartimento di Biologia e Biotecnologie “L. Spallanzani”, Università di Pavia, Pavia, Italy; School of Biological Sciences, Monash University, Clayton, VIC Australia

**Keywords:** *Asaia*, Competition, Mosquito, *Wolbachia*

## Abstract

**Background:**

*Wolbachia* is a group of intracellular maternally inherited bacteria infecting a high number of arthropod species. Their presence in different mosquito species has been largely described, but *Aedes aegypti*, the main vector of Dengue virus, has never been found naturally infected by *Wolbachia*. Similarly, malaria vectors and other anophelines are normally negative to *Wolbachia,* with the exception of an African population where these bacteria have recently been detected. *Asaia* is an acetic acid bacterium stably associated with several mosquito species, found as a dominant microorganism of the mosquito microbiota. *Asaia* has been described in gut, salivary glands and in reproductive organs of adult mosquitoes in *Ae. aegypti* and in anophelines. It has recently been shown that *Asaia* may impede vertical transmission of *Wolbachia* in *Anopheles* mosquitoes. Here we present an experimental study, aimed at determining whether there is a negative interference between *Asaia* and *Wolbachia,* for the gonad niche in mosquitoes.

**Methods:**

Different methods (PCR and qPCR, monoclonal antibody staining and FISH) have been used to address the question of the co-localization and the relative presence/abundance of the two symbionts. PCR and qPCR were performed to qualitatively and quantitatively verify the distribution of *Asaia* and *Wolbachia* in different mosquito species/organs. Monoclonal antibody staining and FISH were performed to localize the symbionts in different mosquito species.

**Results:**

Here we provide evidence that, in *Anopheles* and in other mosquitoes, there is a reciprocal negative interference between *Asaia* and *Wolbachia* symbionts, in terms of the colonization of the gonads. In particular, we have shown that in some mosquito species the presence of one of the symbionts prevented the establishment of the second, while in other systems the symbionts were co-localized, although at reduced densities.

**Conclusions:**

A mutual exclusion or a competition between *Asaia* and *Wolbachia* may contribute to explain the inability of *Wolbachia* to colonize the female reproductive organs of anophelines, inhibiting its vertical transmission and explaining the absence of *Wolbachia* infection in *Ae. aegypti* and in the majority of natural populations of *Anopheles* mosquitoes.

**Electronic supplementary material:**

The online version of this article (doi:10.1186/s13071-015-0888-0) contains supplementary material, which is available to authorized users.

## Background

Most insects harbour mutualistic microorganisms that provide specific traits to the host, conferring evolutionary/adaptive advantages. Many beneficial symbionts are acquired by environmental uptakes in every generation, while few are located in the reproductive organs. As a consequence of this localization, they can easily be acquired by the offspring to ensure their transmission to the next insect generations by vertical transmission [1, 2]. To date, only two bacteria have been shown to be located in the reproductive organs in several mosquito species: the alfa-proteobacteria *Wolbachia* and *Asaia* [3].

*Wolbachia* is a group of obligate intracellular bacteria that infect arthropods and nematodes [4]. Within arthropods, *Wolbachia* infects a wide spectrum of insects, reflecting the ability of these bacteria to manipulate host reproduction, favouring their own maternal transmission [5].

These intracellular bacteria were firstly described in *Culex pipiens* [6], but more recently they have been detected in mosquitoes from several genera, including *Aedes*, *Culex*, *Coquillettidia*, and *Mansonia*. Interestingly, until very recently, *Wolbachia* has not been recorded to naturally infect representatives of the genus *Anopheles,* that comprises more than 300 different species, about 60 of whose are malaria vectors [7]. Only very recently, the presence of *Wolbachia* has been detected in a few individuals of a natural small population of *Anopheles gambiae.* Interestingly, the positive mosquito individuals detected in this study clustered into only a few of the examined mating swarms, pinpointing that ecological and environmental factors might play a key role in the establishment of *Wolbachia* infections in the *An. gambiae* host [8].

Similarly, *Wolbachia* has never been detected in natural populations of *Ae. aegypti*, a main vector of Dengue and yellow fever [9].

In the last few years, several studies have revealed the potential of *Wolbachia* to control mosquito-borne diseases. Indeed, it has been shown that the “forced” introduction of some strains of *Wolbachia* in *Ae. aegypti* reduces its competence in transmitting Dengue virus. The mechanisms at the basis of this reduced vectorial capacity have not been elucidated, even though an up-regulation of the mosquito immune response might play a role in this phenomenon [10, 11]. More recently, it has also been proven that *Wolbachia*-infected *Ae. aegypti,* resistant to Dengue virus infection, are able to rapidly replace natural/susceptible populations, thus validating the *Wolbachia*-mediated population-replacement strategy proposed to control mosquito borne diseases [12].

For all these reasons, there were many attempts to infect *Anopheles* mosquitoes with *Wolbachia,* in order to export the novel symbiont-based control strategies to malaria vectors. As pointed out by Hughes & Rasgon [13], the failure of the first attempts, coupled with the apparent lack of natural infection in natural populations of anophelines, suggests that this genus is somehow refractory to *Wolbachia* infection. Nevertheless, in the last few years, trans-infection of cell lines and somatic infection of adults with *Wolbachia* have been achieved, proving that anophelines can also be forced to harbour these bacteria [14]. Recently, a strain of the main Asian malaria vector, namely *Anopheles stephensi*, was stably transinfected by embryonic microinjection of the *w*AlbB *Wolbachia* strain derived from *Aedes albopictus* [15].

*Asaia* is a bacterium stably associated with numerous mosquito species, including several anophelines, often being the dominant microorganism of the mosquito microbiota. This acetic acid symbiont localises in the gut, salivary glands and reproductive organs of adult mosquitoes. *Asaia* is horizontally transmitted through an oral route during feeding both in pre-adult and adult stages and through a venereal pattern during mating in adults [16, 17]. Moreover, *Asaia* is vertically transmitted from mother to progeny indicating that it may rapidly spread in natural mosquito populations [17, 18].

Both *Wolbachia* and *Asaia* have been proposed as promising microorganisms for the development of symbiont-based control methods to contrast vector-borne diseases [3, 19].

Here we show that the abundances of *Wolbachia* and *Asaia* are negatively related in the reproductive organs of several mosquito species, with a pattern that approximate a mutual exclusion, particularly in anophelines. This provides a possible explanation for the absence of *Wolbachia* in natural populations of *Anopheles*. For simplicity, the phenomenon investigated in this work, *i.e.* the interference between the two symbionts for the colonization of the mosquito gonad niche, will be referred to as ‘competition’.

## Methods

### Mosquito strains

Insectary reared strains: The *An. stephensi* colony (Liston strain) has been maintained for 4 years in the insectary at the University of Camerino (Unicam). Insects were reared at 29 °C and 85–90 % relative humidity with photoperiods (12:12 Light–dark). Adult insects were maintained in a 5 % sucrose solution, and adult females were fed with mouse blood for egg laying. All animal experiments were carried out according to the Italian Directive 116 of 10/27/92 on the “use and protection of laboratory animals” and in adherence with the European regulation (86/609) of 11/24/86, licence no. 125/94A, issued by the Italian Ministry of Health. The experiments were approved by the ethic committee of the University of Camerino (Protocol number 2/2014).

Larvae were maintained in spring water and fed daily with commercial fish food.

The *An. gambiae* colony was established from samples obtained from the Centre National de Recherche et de Formation sur le Paludisme (CNRFP) in Ouagadougou (Burkina Faso, West Africa) and descend from a wild colony.

The *Ae. albopictus* colony was established from mosquitoes that were field-collected from S. Benedetto del Tronto, Central Italy (42°57′00″N; 13°53′00″E) in 2008. They were maintained in the Unicam insectary in the same condition as described above.

*Cx. quinquefasciatus* species mosquitoes, collected in Hawaii (U.S.A.) in 2008 and provided by the insectary of the Center for Vector Biology, Rutgers University (New Brunswick, NJ, U.S.A.), were maintained in the Unicam insectary in the same condition described above.

The colony of *An. stephensi* stably trans-infected with *w*AlbB *Wolbachia* strain (and the relative control treated with antibiotics to remove *Wolbachia* infection) is the one described in [15], that has been bred for two generations at Unicam insectary before performing the analysis.

The colony of *Ae. aegypti,* stably trans-infected with *w*MelPop *Wolbachia* strain (and the relative control treated with antibiotics to remove *Wolbachia* infection), is the one described in [20], that has been bred for two generations at Unicam insectary before performing the analysis.

Field collected mosquitoes: samples of the species *Ae. albopictus* and *Cx. pipiens* were collected in the following towns in the Umbria region (Central Italy): Bastia Umbra (43°07′71″N; 12°55′51″E); Spello (43°0′3″N; 12°40′44″E); Foligno (42°56′58″N; 12°43′10′E); Collestrada (43°05″10″N; 12°28′47″E); Ponte del Campo (43°05′39″N; 12°25′02″E).

### Mosquito samples

Total DNA was extracted from whole mosquitoes and/or organs (dissected in a drop of sterile 1× PBS using sterile needles under a stereomicroscope) as previously described [21].

### Asaia *and* Wolbachia *detection by specific PCR*

For *Asaia* and *Wolbachia* detection specific oligonuleotides were used: Asafor/Asarev [16] and WolbF (5′- gaagataatgacggtactcac -3′)/ WolbRev2 (5′- gtcagatttgaaccagataga -3′), respectively. PCR was performed in 25-ml reaction, using Dream taq Buffer 1X, dNTPs 0.25 mM, Asafor and Asarev oligos (0.3 mM each), 0.75U DreamTaq Polymerase (Thermo Scientific, Waltham, Massachusetts, USA) and 30 ng of DNA template, measured with a NanoDrop ND-1000 spectrophotometer (Thermo Scientific, Waltham, Massachusetts, USA). An initial denaturation at 94 °C for 3 min was followed by 30 cycles consisting of denaturation at 94 °C for 30 sec, annealing at 60 °C for 30 sec, and extension at 72 °C for 30 sec, concluding with a final extension step of 10 min at 72 °C. The PCR products were electrophoresed on a 1 % agarose gel to determine the presence and general size of the amplified DNA.

### Quantitative PCR (qPCR) detection of native Asaia in different mosquito species

PCR assays were designed to detect DNA of bacteria *Asaia* in organs from 13-day-old *An. stephensi, Ae. albopictus* and *Cx. quinquefasciatus* mosquitoes. genes of the different mosquito species were amplified as housekeeping genes (As-r*ps7*, Ae-*rps7*, CX-*rps3* respectively) to allow the normalization of *Asaia* amount.

Amplification consisted of 50 ng DNA, 1X SybrGreen Master Mix (Fermentas, Vilnius, Lithuania), 200 nM of primers. Primers used to amplify target sequences of *Asaia* 16S rRNA and *rps7* of *An. stephensi* (As-*rps7*) were described in [22], whereas the primers for Cx-*rps3* (*Cx. quinquefasciatus*) and Ae-*rps7* (*Ae. albopictus*) genes are listed below:

- Ae-*rps7*-F: 5′- CGCGCTCGTGAGATCGA-3′
- Ae-*rps7*-R: 5′- GCACCGGGACGTAGATCA-3′
- Cx-*rps3*-F: 5′- AGCGTGCCAAGTCGATGAG-3′
- Cx-*rps3*-R: 5′- ACGTACTCGTTGCACGGATCTC-3′

Reactions were run on a CFX thermocycler (Bio-Rad, Hercules, California, USA) using the following cycling conditions: 1 cycle of 95 °C for 10 min, 40 cycles of 95 °C for 1 min, 60 °C for 1 min, and 74 °C for 30 sec. The PCRs were performed on six pools of organs, from ten individuals, for each mosquito species. Each pool was tested in duplicate. The relative quantity of *Asaia* in the mosquito organs was estimated as gene copy ratio calculating the copy number of *Asaia* 16S rRNA gene/respective housekeeping genes.

The amount of amplified targets was measured using standard curves obtained by eight serial dilutions of specific plasmids for each amplicon (from 2 to 2 × 10^−7^ ng). Standard curves used in the experiments had the following parameters (E = efficiency; R^2 = correlation coefficient):

- *Asaia*: E = 97.2 %; R^2 = 0.995; slope = −3.392
- As-*rps7*: E = 96.2 %; R^2 = 0.999; slope = −3.416
- Ae-*rps7*: E = 99.6 %; R^2 = 1.000; slope = −3.333
- Cx-*rps3*: E = 98.6 %; R^2 = 0.999; slope = −3.356.

### Statistical analysis

The statistical analysis of the amounts of *Asaia*, quantified by qPCR assay, was estimated using the Bio-Rad CFX Manager Software and the GraphPad software (http://www.graphpad.com). Data were obtained from the average of six pools per organ. The value of each pool resulted from the average of two technical replicates that have been compared by the Mann–Whitney test.

### Asaia localization on eggs-surface

*An. stephensi*, *An. gambiae* and *Ae. albopictus* eggs were fixed with 4 % paraformaldehyde for 10 min at 4 °C and washed twice with PBS 1X. The slides were incubated in 1 % Bovine Serum Albumine (BSA) in 1X PBS for 30 min at room temperature and successively for 1 h at 37 °C with anti-*Asaia* monoclonal antibody (patent pending N. MI2012A001529) diluted 1:1000. After three washings in PBS 1X, they were incubated for 30 min at 37 °C with anti-mouse IgG Alexa Fluor 594 conjugate (Invitrogen, Carlsbard, California, USA) diluted 1:100 in 1 % BSA in PBS and washed three times for 10 min with 1X PBS. Slides were mounted with glycerol and visualized by epifluorescent microscopy (Carl Zeiss Axio Observer.Z1, Milan, Italy).

### *Asaia* and *Wolbachia* localization in *Ae. aegypti* by FISH

Midguts and reproductive organs (from female and male mosquitoes) of *Wolbachia*-free and *Wolbachia* trans infected *Ae. aegypti* were dissected and fixed in 4 % paraformaldehyde for 5 min at 4 °C and washed in 1X PBS. The organs were then incubated for 10 min at 37 °C with a 10 mg ml^−1^ pepsin solution and washed twice in a 1X PBS and Tween 20 1 % solution and twice in 1X PBS for 5 min at room temperature. Hybridization was performed in dark conditions for 3 h at 40 °C, with 100 μl of hybridization buffer (0.2X SSC, 40 % formamide, BSA 0.1 mg ml^−1^, salmon sperm 0.1 mg ml^−1^, 10 ng μl^−1^ of each probes). The probes targeting the 16S rRNA gene, were synthesized by Eurofins MWG Operon (Ebersberg, Germany) and consisted of: two *Asaia* probes namely Asaia1.FCy3 (5′-GTGTAAACCGCCTACGCGCC-3′) and Asaia2.FCy3 (5′-ATGGATAGATCCCTACGCGA-3′) [17] 5′-end labeled with Cy3 (absorption/emission at 550/570 nm), and two *Wolbachia* probes namely W2 (5′-CTTCTGTGAGTACCGTCATTATC-3′) [23] and WOL3 (5′- GATTGAAAGAGGATAGAGGA-3′) [24] 5′-end labeled with Cy5 (absorption/emission at 650/670 nm). The probes specifically targeting *Asaia* or *Wolbachia* are non cross-reacting with each other.

After hybridization, the organs were washed in 200 μl of washing buffer (0.2X SSC, 60 % formamide) for 15 min at 40 °C, and subsequently once in 500 μl of 0.1X SSC and twice in 200 μl of 1X SSC for 10 min at room temperature. After that they were washed twice in 1X PBS for 5 min at room temperature. Then 50 ng of DAPI (4′, 6′-diamidino-2-phenylindole) were added, and incubated for 5 min at room temperature. After a wash in 1X PBS for 5 min at room temperature, samples were mounted in anti-fading medium and then observed at a laser-scanning confocal microscope SP2-AOBS (Leica, Wetzlar, Germany). Control experiments involved either treatment of slides with RNase prior probe hybridization or in absence of probe.

### *Asaia*-GFP colonization of reproductive organs of different mosquito species

*An. stephensi*, *Cx. quinquefasciatus* and *Ae. albopictus* mosquitoes were colonized with *Asaia* SF 2.1 (transformed with pHM2-Gfp plasmid) strain isolated from *An. stephensi* [16]. *Ae. albopictus* mosquitoes were also colonized with *Asaia* AA 5.5 (transformed with pHM2-Gfp plasmid) isolated from *Ae. albopictus* Unicam strain.

The mosquitoes were fed with a strain of *Asaia* expressing the Green Fluorescent Protein (namely *Asaia* SF2.1 and AA5.5). These bacteria were grown 24 h at 30 °C in GLY medium (glycerol 25 g/L; yeast extract 10 g/L; pH 5). Cells were harvested by centrifugation, washed three times in 0.9 % NaCl and adjusted to 10^8^ cells per ml^−1^ in 50 ml of H_2_O/5 % (wt/vol) sucrose solution, supplemented with 100 μg·ml^−1^ of kanamycin to avoid the loss of plasmid from bacterial cells. After 4 days of feeding, the cotton pad containing strain SF2.1(Gfp) was removed and replaced with a new sterile 5 % (wt/vol) sucrose solution supplemented with kanamycin. Mosquitoes were sampled and dissected every 2–3 days up to 15 days after initial exposure to the bacterium. Guts and reproductive organs were fixed with 4 % paraformaldeyde for 10 min at 4 °C. The slides were then mounted in glycerol and examined in fluorescent microscopy (Carl Zeiss Axio Observer.Z1, Milan, Italy).

## Results

### *Tissue localization of* Asaia *in different mosquito species*

**Fig. 1:**
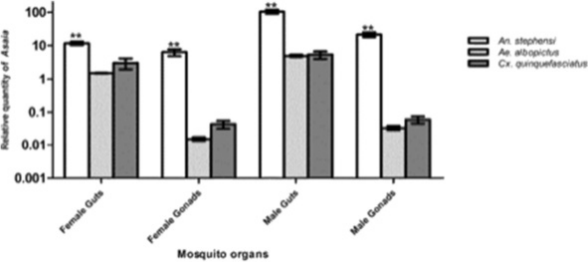
Quantitative detection of *Asaia* in organs of three different lab-reared mosquito species obtained by qPCR. The relative amount of the bacteria is expressed as a ratio of bacterial 16S rRNA and mosquito *rps7* genes (*An. stephensi* and *Ae. albopictus*) or *rps3* gene (*Cx. quinquefasciatus*) copies in a logarithmic scale. Abundance results from the mean±SEM of six pools (10 organs) for each species. Statistically significant differences are represented by asterisks (*p*<0.01) as determined by multiple comparisons using Mann Whitney test

Firstly, by specific PCR assays we have investigated the tissue distribution of *Asaia* in laboratory-reared strains of *Ae. albopictus* and *Cx. quinquefasciatus*, two species of mosquitoes that are naturally infected with *Wolbachia*. Interestingly, while *Asaia* was found in the midguts of these mosquitoes, it was never detected in the reproductive organs (Table 1). This represents a remarkable difference if compared with *Asaia* distribution in tissues of mosquito species that are not infected with *Wolbachia,* such as *An. gambiae, An. stephensi*, *Ae. aegypti*, where *Asaia* has been constantly found in the reproductive organs and salivary glands, in addition to the midgut [25]. We have also evaluated the circulation of *Asaia* in different mosquito organs of *An. stephensi*, *Ae. albopictus* and *Cx. quinquefasciatus* by quantitative PCR. As shown in Fig. 1, occasionally, it was possible to detect the presence of *Asaia* in the reproductive organs of the two latter species, but in a very limited amount compared with that present in the midguts and in the reproductive organs of *An. stephensi*. Indeed, the differences in the *Asaia* load in the midguts and gonads of *Ae. albopictus* and *Cx. quinquefasciatus*, as revealed by qPCR, were evident and statistically significant. Of course, we cannot rule out the possibility that, when dissecting organs for the molecular analysis, *Asaia* bacterial cells located in the guts have contaminated the reproductive organs.

**Table 1:** PCR detection of Asaia in lab-reared mosquito species. Six pools of different organs from ten individuals per each mosquito species were analysed by Asaia-specific PCR assay

	*Anopheles gambiae*	*An. stephensi*	*Aedes aegypti*	*Ae. albopictus*	*Culex quinquefasciatus*
Male Guts	100 % Positive	100 % Positive	100 % Positive	100 % Positive	75 % Positive
Female Guts	100 % Positive	100 % Positive	100 % Positive	100 % Positive	75 % Positive
Male Gonads	100 % Positive	100 % Positive	100 % Positive	Negative	50 % Positive
Female Gonads	100 % Positive	100 % Positive	100 % Positive	Negative	Negative

To further corroborate the null or very limited circulation of *Asaia* in the reproductive organs of *Wolbachia* infected mosquitoes, we have analysed *Ae. albopictus* and *Cx. pipiens* mosquitoes field collected in Italy. A high proportion of these mosquitoes did not host *Asaia* (63 % and 85 % respectively), as revealed by specific PCR assay on whole mosquitoes (Additional file 1: Table A).

### *Asaia* localization on eggs-surface

**Fig. 2:**
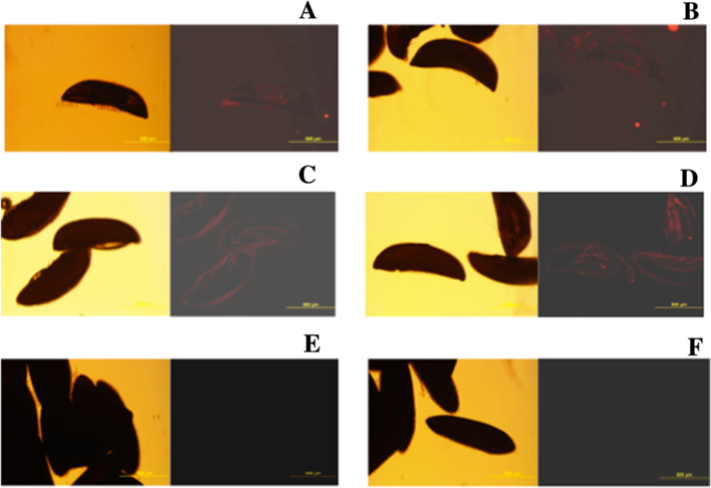
Detection of *Asaia* sp. on different species of mosquito eggs by IFA with anti-*Asaia* mAb. Eggs of *An. stephensi* (**a,b**), *An. gambiae* (**c,d**) and *Ae. albopictus* (**e,f**) are shown. **a**, **c** and **f** represent the treatment with secondary antibody only. Red signal shows the presence of *Asaia* on the surface of the eggs of *An. stephensi* and *An. gambiae* (**b,d**) while no signal was detected on the eggs of *Ae. albopictus* (**e**). Phase contrast images are shown in the boxed areas of the panel

The specific localization of *Asaia* in different tissues of mosquitoes *Ae. albopictus* and *Cx. quinquefasciatus* may explain the inability of these mosquitoes to vertically transmit *Asaia*, differently to what has been shown in *An. gambiae* [17]. To further investigate this aspect, we used a specific antibody to localize *Asaia* on the surface of eggs produced by different mosquito species, some naturally hosting *Wolbachia* and some not. Antibody-based staining revealed the presence of *Asaia* on the surface of eggs of *An. stephensi* and *Ae. aegypti,* while no signal was detected on the eggs of *Ae. albopictus* and *Cx. quinquefasciatus* (Fig. 2). The results of these experiments are congruent with those of tissue localization, reinforcing the hypothesis that the presence of *Wolbachia* prevents the establishment of *Asaia* within the reproductive organs of *Ae. albopictus* and *Cx. quinquefasciatus* (and vice-versa in anopheline mosquitoes and *Ae. aegypti*).

### *Asaia*-GFP colonization of midguts and reproductive organs of different mosquito species

**Fig. 3:**
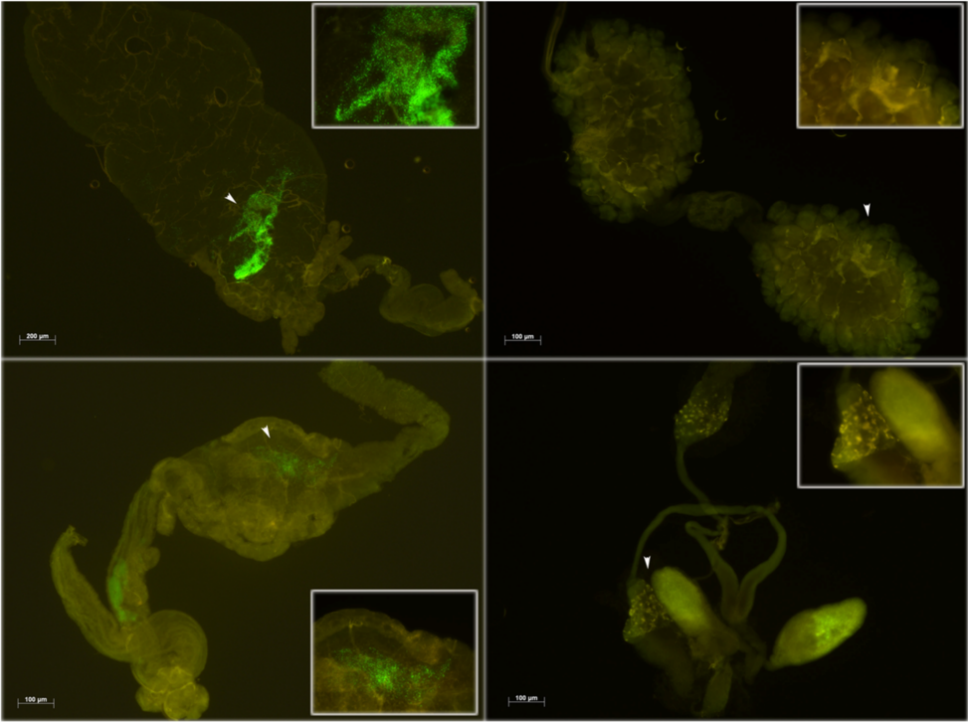
*Asaia*-GFP colonization in different organs of *Cx. quinquefasciatus*. Left images show guts of female (top) and male (bottom) *Cx. quinquefasciatus* mosquitoes analyzed after colonization with *Asaia*-GFP provided with sugar solution. Arrows indicate the localization of main colonization (this area has been magnified in the square). Right images show gonads of female (top) and male (bottom) mosquitoes analyzed after colonization with *Asaia*-GFP. Even in the magnified area no signal of colonization is detected

To further investigate the hypothesis of a microbial competition between *Asaia* and *Wolbachia* within the reproductive organs of mosquitoes, a series of experiments has been performed using a strain of *Asaia* expressing the green fluorescent protein (GFP), provided to the mosquitoes with sugar meal to obtain body colonization. We focused our attention on midguts and gonads. In *Ae. albopictus* and *Cx. quinquefasciatus*, the colonization with *Asaia*-GFP strain was limited to the midguts of the examined specimens, that were colonized in the 81 % and 80,3 % of the cases respectively, while no colonization was detected in the reproductive organs in any of the 60 specimens analyzed for each species (Fig. 3), (Additional file 2: Table B). These findings are just the opposite with those previously reported in *An. gambiae*, *An. stephensi* and *Ae. aegypti* where the colonization has been repeatedly observed in both midguts and reproductive organs [3] and confirmed by the same analysis in 60 specimens *An. stephensi* mosquitoes, that has been used as control, that were colonized in 86 % of the guts and in 30 % of the reproductive organs (Additional file 2: Table B).

### *Asaia*-GFP colonization of reproductive organs of a strain of *Ae. aegypti* stably trans-infected with *Wolbachia w*MelPop

**Fig. 4:**
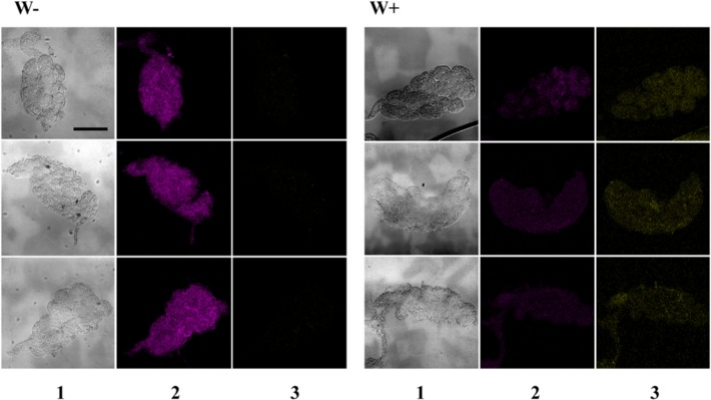
*Asaia* and *Wolbachia* detection in gonads of *w*MelPop infected (W+) and uninfected (W^−^) *Ae. aegypt*i mosquitoes. Detection is shown through interferential contrast microscopy (column 1) and after whole mount *in situ* hybridization with *Asaia* (in magenta, column 2) and *Wolbachia* (in yellow, column 3) specific probes. Bar corresponds to 120 um

The colonization experiments with *Asaia-GFP* were then extended to a strain of *Ae. aegypti* stably trans-infected with a *Wolbachia* from *Drosophila melanogaster*, named *w*MelPop [26]. In these *Wolbachia*-infected *Ae. aegypti* mosquitoes, the *Asaia* colonization rate was similar to that of the control, represented by the wild type *Wolbachia-*uninfected *Ae. aegypti* from the same strain (100 % vs 100 % in the guts, 36 % vs 39 % in the reproductive organs) (Additional file 3: Table C). Even though, these data do not seem to support a competition between the two microbes, the trans-infected strain and the control strain of *Ae. aegypti* have also been analysed by FISH assay to quantify the rate of infection. By comparing the intensity of the signal in *w*MelPop infected and uninfected mosquitoes, it was fairly evident that in the presence of *Wolbachia*, the amount of *Asaia* in the reproductive organs was lower than that in the absence of *Wolbachia* (Fig. 4).

### *Asaia*-GFP colonization of reproductive organs of a strain of *An. stephensi* stably trans-infected with *Wolbachia w*AlbB

The colonization experiments were extended also to a strain of *An. stephensi* that has been trans-infected with a *Wolbachia* strain from *Ae. albopictus,* named *w*AlbB. The control was represented by the wild type *Wolbachia-*uninfected strain of *An. stephensi*. In both the *Wolbachia* infected and uninfected mosquitoes the guts were constantly colonised by *Asaia* (100 % in both strains), while in the reproductive organs no colonization was detected in *Wolbachia*-positive mosquitoes. On the other hand, colonization was observed in the *Wolbachia* uninfected mosquitoes, in 76 % of the ovaries and 20 % of the testis, out of 25 specimens examined per each organ (Table 2).

**Table 2:** Colonization experiments with Asaia-GFP of a strain of Anopheles stephensi stably trans-infected with Wolbachia (W+) and a wild type Wolbachia-uninfected An. stephensi strain (W^−^). Asaia-GFP was provided to mosquitoes with the sugar meal. Twenty five organs were analysed per each strain and relative positivity is reported

	*An. stephensi* W+	*An. stephensi* W^−^
Guts ♂	100 %	100 %
Guts ♀	100 %	100 %
Reproductive organs ♂	0 %	20 %
Reproductive organs ♀	0 %	76 %

## Discussion

Despite its common presence in arthropods, including several mosquito species, *Wolbachia* has not been detected in anophelines, with just one recent description in a few mating swarms from a small population of *An. gambiae* [8]. To verify possible causes of this phenomenon, we have tested the hypothesis that microbial competition may contribute to explain the absence of *Wolbachia* infection within members of the *Anopheles* genus. In particular, we have tested the competition that may occur at the reproductive organs, since *Wolbachia* is a well-known manipulator of host reproduction that exploits effects exerted at the level of the gonads to increase its own fitness [27, 28]. Our hypothesis is that microorganisms that should in some way reduce the amount of *Wolbachia* within the gonads would also reduce the overall capacity of *Wolbachia* to induce reproductive manipulations, such as cytoplasmic incompatibility (CI), thus reducing the capacity of *Wolbachia* to spread (or to be maintained) into a population.

We have focused our attention on *Asaia*, the only other bacterium known to localize in the mosquito’s reproductive organs, and thus a potential competitor of *Wolbachia* for these anatomical niches.

Our overall results support the hypothesis that a competition between the two bacteria occurs at the reproductive organs, and this competition is particularly evident in the ovaries, that are essential not only for the maternal vertical transmission of *Wolbachia*, but also for determining phenomena like the rescuing of the sperm in CI, and thus for ensuring the differential fitness of *Wolbachia* infected VS uninfected females.

First, we have shown that in *Cx. quinquefasciatus* and *Ae. albopictus* (that are naturally infected by *Wolbachia*), *Asaia* infects the gut, but does not infect the reproductive organs. Our previous works, however, showed that mosquito-species naturally uninfected with *Wolbachia* (i.e. *An. gambiae*, *An. stephensi*, *Ae. aegypti*) host *Asaia* in the reproductive organs, as well as in other anatomical districts [17, 29, 30].

Secondly, the IFA-based comparative investigation, aimed at detecting the presence of *Asaia* on the eggs surface of *An. gambiae*, *An. stephensi* and *Ae. albopictus*, showed that only this latter species, the only one naturally infected with *Wolbachia*, did not carry *Asaia* on the egg surface. This is congruent with the absence of *Asaia* in the reproductive organs in *Ae. albopictus*, which is thus to be regarded as a dead-end host for these bacteria, in contrast to what was observed in *An. stephensi* (this study) and *An. gambiae* [17], where egg-smearing appears as the mechanism for the vertical transmission of these bacteria.

Thirdly, the colonization with an *Asaia*-GFP strain was detectable only in the guts, and not in the gonads, in *Cx. quinquefasciatus* and *Ae. albopictus,* while this strain of *Asaia* has already been shown to be very effective in the colonization of the reproductive organs in *An. gambiae*, *An. stephensi*, *Ae. aegypti* [3, 25].

Finally, we showed that *Asaia*-GFP is able to colonize two *Wolbachia* artificially and stably infected strains of mosquito (*Ae. aegypti* and *An. stephensi)*. In both cases we observed that an unnatural presence of *Wolbachia* in guts and reproductive organs does not alter the ability of *Asaia*-GFP to efficiently colonize the guts of recipient mosquitoes. Conversely, the presence of *Wolbachia* in the reproductive organs may strongly influence the ability of *Asaia*-GFP to efficiently colonize the reproductive organs of trans-infected mosquitoes in respect to the control strains. In *Ae. aegypti* we detected a similar percentage of colonized mosquitoes between *Wolbachia* infected and uninfected mosquitoes, although *Asaia* has been shown to be less abundant in the reproductive organs of *Wolbachia*-positive mosquitoes, compared to *Wolbachia*-negative ones.

In *An. stephensi* this difference between *Wolbachia*-positive and -negative mosquitoes was stronger, in that no colonization was observed in the reproductive organs from *Wolbachia-*positive mosquitoes*,* while colonization of reproductive organs in *Wolbachia-*negative insects was detected in about 75 % of the specimens tested. The above reported differences between *Ae. aegypti* and *An. stephensi* may be due to intrinsic species-specific factors and/or to the fact that the two species were infected with different *Wolbachia* strains: *Ae. aegypti* with one from *Drosophila*, the virulent strain *w*MelPop of *Wolbachia*, and *An. stephensi* with one from *Ae. albopictus*.

All these evidences taken together, underline a role of *Wolbachia* in preventing some mosquito species from a stable and successful *Asaia* infection in the gonads. On the other hand, our data seem to support a role of *Asaia* in the inability of *Wolbachia* to infect anopheline mosquitoes.

*Asaia* seems to exert its action specifically at the level of reproductive organs and particularly in ovaries suggesting that these two bacteria may compete for the same anatomical niche or infection routes in the host reproductive organs so that in the presence of *Asaia*, *Wolbachia* cannot colonize these organs, or that colonization is in some way limited. As already emphasized, a limited colonization of male and female gonads is likely to reduce the strength of reproductive manipulations such as CI, and, in the case of the female gonad, a limited (or absent) colonization will obviously reduce the efficacy of vertical transmission.

These conclusions are congruent with the results of a recent study described by Hughes and collaborators that have shown that *Asaia* may impede vertical transmission of *Wolbachia* in *Anopheles*. [31]. Conversely, it has recently been demonstrated also that the introduction of *w*AlbB in *An. stephensi* reduces female fecundity and causes a minor decrease in male mating competiveness [32].

## Conclusions

Symbiont competition and in particular the competition for anatomical niches such as the gonads appears an important but under-investigated phenomenon, that is likely to have an impact on the establishment of symbioses in insects.

Although we cannot exclude that other microorganisms may take part in the “competition for gonads”, it is reasonable to assume that the phenomenon is not necessarily based on a ‘complete absence’ VS a ‘complete presence’ of particular symbionts. Nevertheless, our findings update the current knowledge on mosquito symbiosis and may have important implications for the development of symbiont-based control of mosquito-borne diseases, a research area where both *Asaia* and *Wolbachia* are regarded as important candidates.

## Additional files

Additional file 1:
**Table A.** Assessment of *Asaia* and *Wolbachia* circulation in field collected mosquitoes. Percentage of field collected mosquitoes naturally infected with *Asaia* and *Wolbachia* was detected by specific PCR assay. The total number of mosquito examined was 104. Additional file 2:
**Table B.** *Asaia*-GFP tissue specific colonization. Sixty individuals of three different mosquito species were provided with sugar meal enriched with *Asaia*-GFP. Percentages of colonized guts and gonads are reported. Additional file 3:
**Table C.** Colonization experiments with *Asaia-*GFP in a strain of *Ae. aegypti* stably trans-infected with a *Wolbachia* (W+) and wild type *Wolbachia-*uninfected *Ae. aegypti* strain (W^−^). Sixty individuals of both mosquito species were provided with sugar meal enriched with *Asaia*-GFP. Percentages of colonized guts and gonads are reported.
