# Supplementary material for: Mutual exclusion of Asaia and Wolbachia in the reproductive organs of mosquito vectors
Source: Parasit Vectors. 2015 May 17;8:278. doi: 10.1186/s13071-015-0888-0 (PMC4445530; doi:10.1186/s13071-015-0888-0)
Supplement: Additional file 1: — Table A. Assessment of Asaia and Wolbachia circulation in field collected mosquitoes. Percentage of field collected mosquitoes naturally infected with Asaia and Wolbachia was detected by specific PCR assay. The total number of mosquito examined was 104. [file 13071_2015_888_MOESM1_ESM.doc]

**Additional file 1**

Table A

|  | **PCR positive for *Asaia*** | **PCR positive for *Wolbachia*** | **Samples simultaneously positive for both bacteria** |
| --- | --- | --- | --- |
| *Aedes albopictus* | **37,36%**  (38% of the females)  (36,6% of the males) | **83,5%**  (88% of the females)  (78% of the males) | **28.57%**  (34% of the females)  (21.95% of the males) |
| *Culex pipiens* | **15,4%**  (22.22% of the females)  (0% of the males) | **92,3%**  (100% of the females)  (75% of the males) | **15,4%**  (22.22% of the females)  (0% of the males) |

**Assessment of *Asaia* and *Wolbachia* circulation in field collected mosquitoes.** Percentage of field collected mosquitoes naturally infected with *Asaia* and *Wolbachia* was detected by specific PCR assay. The total number of mosquito examined was 104.
