# Supplementary material for: Mutual exclusion of Asaia and Wolbachia in the reproductive organs of mosquito vectors
Source: Parasit Vectors. 2015 May 17;8:278. doi: 10.1186/s13071-015-0888-0 (PMC4445530; doi:10.1186/s13071-015-0888-0)
Supplement: Additional file 2: — Table B. Asaia-GFP tissue specific colonization. Sixty individuals of three different mosquito species were provided with sugar meal enriched with Asaia-GFP. Percentages of colonized guts and gonads are reported. [file 13071_2015_888_MOESM2_ESM.doc]

**Additional file 2**

Table B

|  | *Aedes albopictus* | *Culex quinquefasciatus* | *Anopheles stephensi* |
| --- | --- | --- | --- |
| Guts | 81% | 80,3% | 86% |
| Gonads | 0% | 0% | 31.5% |

***Asaia*-GFP tissue specific colonization.** Sixty individuals of three different mosquito species were provided with sugar meal enriched with *Asaia*-GFP. Percentages of colonized guts and gonads are reported.
