# Supplementary material for: Mutual exclusion of Asaia and Wolbachia in the reproductive organs of mosquito vectors
Source: Parasit Vectors. 2015 May 17;8:278. doi: 10.1186/s13071-015-0888-0 (PMC4445530; doi:10.1186/s13071-015-0888-0)
Supplement: Additional file 3: — Table C. Colonization experiments with Asaia-GFP in a strain of Ae. aegypti stably trans-infected with a Wolbachia (W+) and wild type Wolbachia-uninfected Ae. aegypti strain (W−). Sixty individuals of both mosquito species were provided with sugar meal enriched with Asaia-GFP. Percentages of colonized guts and gonads are reported. [file 13071_2015_888_MOESM3_ESM.doc]

**Additional file 3**

Table C

|  | *Aedes aegypti* W+ | *Aedes aegypti* W- |
| --- | --- | --- |
| Guts | 100% | 100% |
| Gonads | 36% | 39% |

**Colonization experiments with *Asaia-*GFP in a strain of *Ae. aegypti* stably trans-infected with a *Wolbachia* (W+) and wild type *Wolbachia-*uninfected *Ae. aegypti* strain (W-).** Sixty individuals of both mosquito species were provided with sugar meal enriched with *Asaia*-GFP. Percentages of colonized guts and gonads are reported.
